# Cytochrome P450-2D6 activity in people with codeine use disorder

**DOI:** 10.1038/s41397-023-00319-6

**Published:** 2023-11-09

**Authors:** Mark R. C. Daglish, Sarah R. Reilly, Sam Mostafa, Cameron Edwards, Thomas M. O’Gorman, Jeremy S. Hayllar

**Affiliations:** 1 Alcohol & Drug Service, The Prince Charles Hospital, Metro North Health, Brisbane, QLD Australia; 2 Hospital Alcohol & Drug Service, Royal Brisbane and Women’s Hospital, Metro North Health, Brisbane, QLD Australia; 3 Faculty of Medicine, The University of Queensland, Brisbane, QLD Australia; 4 myDNA Life, Australia Ltd, South Yarra, VIC Australia; 5 Centre for Medicine Use and Safety, Monash University, Parkville, VIC Australia

**Keywords:** Addiction, Genotype

## Abstract

Compound-analgesics containing codeine (CACC) have been a common source of codeine for people seeking opioid replacement therapy (ORT) for codeine use disorder (CUD). Our previous work demonstrated no relationship between pre-treatment CACC and ORT buprenorphine doses; we hypothesised that CYP2D6 activity would partially account for this disconnection. One hundred six participants with CUD were compared to a published population sample of 5408 Australian patients. Mean age of participants with CUD at treatment entry was 35 years, with mean 6.1 years duration of CUD. Mean codeine dose was 660 mg/day (range 40–2700 mg). Mean calculated CYP2D6 activity scores were significantly higher in the codeine group (CUD 1.65 + 0.63 vs. Gen pop 1.39 + 0.65, Wilcoxon *W* = 347,001, *p* < 0.001). Pre-treatment CACC dose weakly predicted sublingual buprenorphine doses overall; there was a stronger relationship within ultrarapid metabolisers. While normal and ultrarapid metabolisers of codeine were more likely to have a diagnosis of CUD, poor or intermediate CYP2D6 metaboliser status may protect against CUD.

## Introduction

Codeine has historically been considered a “weak opioid” [1] with combination analgesics containing codeine (CACC) readily accessible over the counter in many countries. In 2016 an estimated 3.6% of Australians had used codeine, frequently CACC, for non-medical purposes [2]. This has been linked with significant morbidity & mortality resulting from excessive consumption of ibuprofen and paracetamol [3, 4]. In 2019, that figure fell to 1.5%, reflecting the rescheduling of CACC to prescription-only in Australia on 1 February 2018 [5]. Despite this, codeine remains heavily prescribed in Australia and internationally, with alcohol and other drug (AOD) services continuing to treat patients presenting with primary codeine use disorder (CUD) [6].

The limited studies to date have highlighted the difficulty with opioid replacement therapy (ORT) dose estimation in patients with CUD. Codeine intake at presentation is a poor predictor of buprenorphine requirement with doses of ORT required being higher than expected [7]. The Oral Morphine Equivalent of codeine is typically described as about 7:1, thus each 7 mg of codeine ingested produces about 1 mg morphine [8]. However, codeine is an inactive prodrug; its analgesic efficacy is reliant on metabolism to morphine by O-demethylation through the cytochrome P450-2D6 (CYP2D6) enzyme, which is subject to significant genetic variation [9].

Variations in the gene encoding the CYP2D6 enzyme can significantly affect enzymatic function and the metabolism of a range of substrate opioid medications including codeine, oxycodone and tramadol. The classification of individuals by CYP2D6 status has evolved, with the current system based on assigning activity scores ranging from 0 to 1 for each allele present, with a score of 1 equating to “normal” activity, scores less than 1 for reduced activity and 0 meaning no activity or non-functional. An individual’s total genotype activity score is the sum of all alleles ranging from 0, with two or more non-functioning alleles, to 3 or more when multiple functional copies are present [9]. While various cut-off values have been proposed, the current consensus is that scores greater than 2.25 are classified as ultrarapid metabolisers (UM), scores of 1.25 to 2.25 as normal metabolisers (NM), scores less than 1.25 but greater than 0 as intermediate (IM) and scores of 0 as poor metabolisers (PM) [9].

CYP2D6 metaboliser status has clinical implications for codeine, with effects on analgesic response [10] and UM at increased risk of morphine toxicity [11]. CYP2D6 variations may also increase the likelihood of individuals developing CUD and influence their opioid replacement requirement when presenting for treatment. There is data on the prevalence of CYP2D6 activity in various populations and known ethnic variation [12, 13]. Approximately 6% of an Australian patient sample were poor metabolisers and nearly 3% ultrarapid metabolisers [12]. To date, there have been no published results of CYP2D6 activity in people who have developed CUD.

The present study’s primary hypothesis was that people who are codeine NM and UM are overrepresented among those who have commenced ORT for CUD compared to the general population. The secondary hypotheses were that CYP2D6 metaboliser status might predict doses of ORT; that CYP2D6 status would predict doses of codeine used; that codeine doses prior to treatment would predict buprenorphine doses required; and that CYP2D6 activity would influence the relationship between codeine taken prior to admission to treatment and ORT buprenorphine doses required.

This study will build understanding about the range of risks to which UM individuals may be exposed. If the research hypotheses are confirmed, it will add to the range of precautions which need to be taken before codeine is prescribed; not only does UM confer an increased risk of toxicity, but also an increased risk of developing CUD.

## Methods

### Design

This was a cross-sectional study comparing the frequencies of CYP2D6 metaboliser phenotype groups in a sample of people with CUD seeking ORT, with previously published frequencies in an Australian population [12].

### Participants

Participants were recruited between July 2019 and March 2022 from four sites of a large metropolitan Alcohol & Drug Service in Brisbane, Australia. People with CUD receiving ORT or withdrawal management were invited to participate. Inclusion criteria were age 18 or older, opioid use disorder with codeine the primary drug of concern as determined by the treating clinical team based on clinical assessment and urine drug testing, and able to provide informed consent. Use of other substances was not an exclusion criterion, but codeine had to be the primary drug of concern at the time of presentation for treatment. Eligible participants received a AU$30 supermarket voucher and provided a buccal cell swab from which DNA was extracted. The control sample of 5408 Australians was drawn from previously published work [12].

Approval for the study was obtained from the Human Research Ethics Committee of The Prince Charles Hospital, Brisbane, Australia covering the 4 clinic sites (study approval number 51449).

### Sample testing and genotype to phenotype translation

CYP2D6 genotyping was performed by GenSeq Labs (a subsidiary of MyDNA Life Australia Limited, Melbourne, Australia). Genomic DNA was extracted from a buccal swab sample and SNP genotyping was performed using open array technology (Life Technologies QuantStudio 12K, Thermo Fisher, Waltham MA, USA). CYP2D6 copy number was established by real time PCR (QuantStudio 6), allowing for quantification of up to 4 copies. 3D PCR (QuantStudio 3D) was used to determine which allele was duplicated. The following clinically actionable CYP2D6 alleles were tested: *2, *3, *4, *5, *6, *7, *8, *9, *10, *12, *14A, *14B, *17, *29, *36, *41 and *xN. The CYP2D6 genotype to phenotype translation followed the Clinical Pharmacogenetics Implementation Consortium and Dutch Pharmacogenetics Working group consensus recommendations [14].

### Measures

Using self-report and the medical notes, data were collected for participant demographics, maximum doses and duration of codeine use prior to treatment, smoking status, other medication use, and treatment measures. The maximum buprenorphine or methadone doses and duration of treatment were collected for all participants. For participants who commenced ORT we also collected stabilisation dose, operationalised as the first daily dose that did not require adjustment for at least 1 week. Dose titration was based on clinical assessment focused on client subjective experience of the absence of codeine withdrawal symptoms and self-reported cessation of use of, and craving for, codeine according to local treatment guidelines [15].

### Data analysis

Data were analysed using R in RStudio for Windows [16, 17]. Demographics were summarised with descriptive statistics. The genotype analysis plan including power calculations was registered with OSF prior to data analysis [18]. Genotype results for the previously published sample were originally reported in the (now superseded) five phenotype groups (ultra-rapid, normal, low normal, intermediate and poor). These were re-mapped to the current classification. For the primary analysis the sample genotype groups were collapsed into a binary phenotype split of metabolisers (Ultrarapid + Normal) and non-metabolisers (Intermediate + Poor) and the proportions in the CUD and general population compared with a Chi-squared test and odds ratios. A secondary ordinal analysis was undertaken across all four phenotypes using a non-parametric Wilcoxon signed rank test and ordinal logistic regression; the Wilcoxon test was also repeated using allele activity scores. A sequence of regression analyses was used to test the secondary hypotheses that codeine doses predicted ORT doses and the interaction between CYP2D6 status and codeine dose on ORT dose. Linear regression models were chosen as any relationship between codeine and buprenorphine was expected to be approximately linear over the relevant dose range of codeine and buprenorphine. Buprenorphine is a mu-opioid partial agonist [15] so we hypothesised the relationship would be logarithmic over the entire dose range, but did not expect people to present for treatment of CUD on low doses of codeine or require low doses of buprenorphine.

## Results

A total of 106 individual participants with CUD were recruited across the four sites. Two participants were inadvertently recruited twice and supplied two buccal swabs for DNA analysis; their results were identical, and the duplicates were removed from further analysis. Participant demographics and treatment choices are presented in Table 1.

**Table 1:** Participant Demographics and summary.

Demographics				CUD group	
Gender	Male			58 (55%)	
	Female			48 (45%)	
Age			Mean ± sd	36.4 ± 8.6 years	
Smoking	Non-smoker			35 (35%)	
	Ex-smoker			10 (10%)	
	Current smoker			56 (55%)	
Codeine Use	Duration		Mean ± sd	6.0 ± 6.5 years	
	Max daily dose	Overall	Mean ± sd	660 ± 487 mg/day	
			Range	40–2700 mg/day	
		PM	Mean ± sd	750 ± 397 mg/day	^a^*F*_(3,93)_ = 0.88, *p* = 0.45
		IM	Mean ± sd	639 ± 508 mg/day	
		NM	Mean ± sd	698 ± 507 mg/day	
		UM	Mean ± sd	408 ± 175 mg/day	
Treatment choice	Buprenorphine	Withdrawal only		5 (5%)	
		ORT		95 (91%)	
	Methadone	ORT		4 (4%)	
ORT doses	Buprenorphine ORT stabilisation doses		Median, IQR	16, 8–20 mg/day	
			Range	2–40 mg/day	
	Buprenorphine ORT max doses		Median, IQR	20, 16–28 mg/day	
			Range	2–40 mg/day	
	Buprenorphine withdrawal only max doses^b^		Median, IQR	1, 0.6–6 mg/day	
			Range	0.3–10 mg/day	
	Methadone ORT max doses		Range	35–160 mg/day	

*ORT* opioid replacement therapy, *PM* poor metaboliser, *IM* intermediate, *NM* normal, *UM* ultrarapid metaboliser.

^a^Result from one-way ANOVA.

^b^One participant used transdermal buprenorphine patches only.

**Fig. 1:**
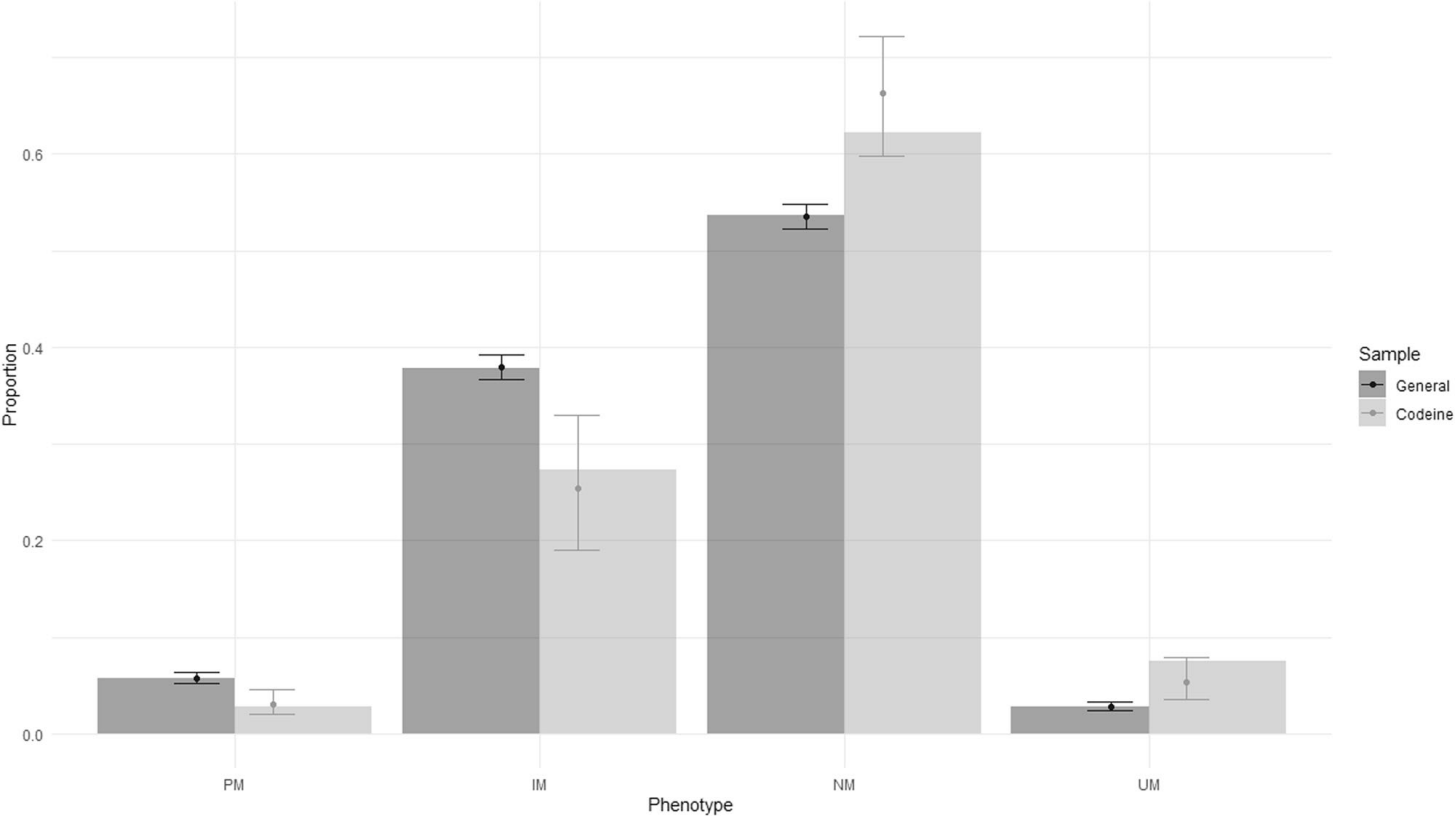
Proportions in CPIC phenotype groups. Filled bars represent observed proportions. Error bars represent predicted proportions from ordinal logistic regression with 95% CI. General general population, Codeine codeine use disorder group, PM poor metaboliser, IM intermediate metaboliser, NM normal metaboliser, UM ultrarapid metabolizer.

Genotype results are shown in Table 2. All DNA samples yielded a valid genotype that could be matched to a phenotype group using CPIC guidelines [9, 14]. Allele frequencies in the CUD sample did not significantly deviate from Hardy-Weinberg Equilibrium using the same methods as the population sample [12] derived from a previously established method [13]. Data from the control sample [12] were converted to the same revised CPIC phenotype groups. There was a significant increase in the proportion of NM + UM in CUD compared to the general population (70% vs. 56%, *Χ*^2^ = 7.1, df = 1, *p* = 0.008; OR = 1.78 95% CI: 1.18–2.75). There was also a significant shift in proportions across the four CPIC groups towards higher CYP2D6 activity phenotypes in the CUD group compared to the general population sample (PM 2.8% CUD vs. 5.8% gen. pop., IM 27.4% vs. 37.9%, NM 62.3% vs. 53.6%, UM 7.5% vs. 2.8%, Wilcoxon *W* = 333,260, *p* < 0.001), as shown in Fig. 1. Testing the same effect with ordinal logistic regression yielded a calculated odds ratio of 1.61 (95% CI: 1.21–2.15, *p* < 0.01) meaning that being in the CUD group conferred a 60% increase in the odds of being in a higher activity phenotype group. Expected proportions in each group were calculated from the ordinal logistic regression and are also shown in Fig. 1 for comparison. Mean calculated CYP2D6 activity scores were also significantly higher in the CUD group (CUD 1.65 ± 0.63 vs. Gen pop 1.39 ± 0.65, Wilcoxon *W* = 347,001, *p* < 0.001).

**Table 2:** Genotype results.

	CPIC grouping	Binary grouping	Genotypes
Poor metaboliser	3 (2.8%)	32 (30%)	*4/*4, *4/*6
Intermediate metaboliser	29 (27.4%)		*1/*3, *1/*4, *1/*5, *2/*3, *2/*4, *2/*5, *2/*6, *4/*9, *9/*41
Normal metaboliser	66 (62.3%)	74 (70%)	*1/*1, *1/*10, *1/*2, *1/*41, *1/*41×2, *1/*9, *2/*2, *2/*41, *2/*9
Ultrarapid metaboliser	8 (7.5%)		*1/*1 × 2, *1/*2 × 2, *1 × 2/*41, *2/*2 × 2, *2 × 3/*4

**Fig. 2:**
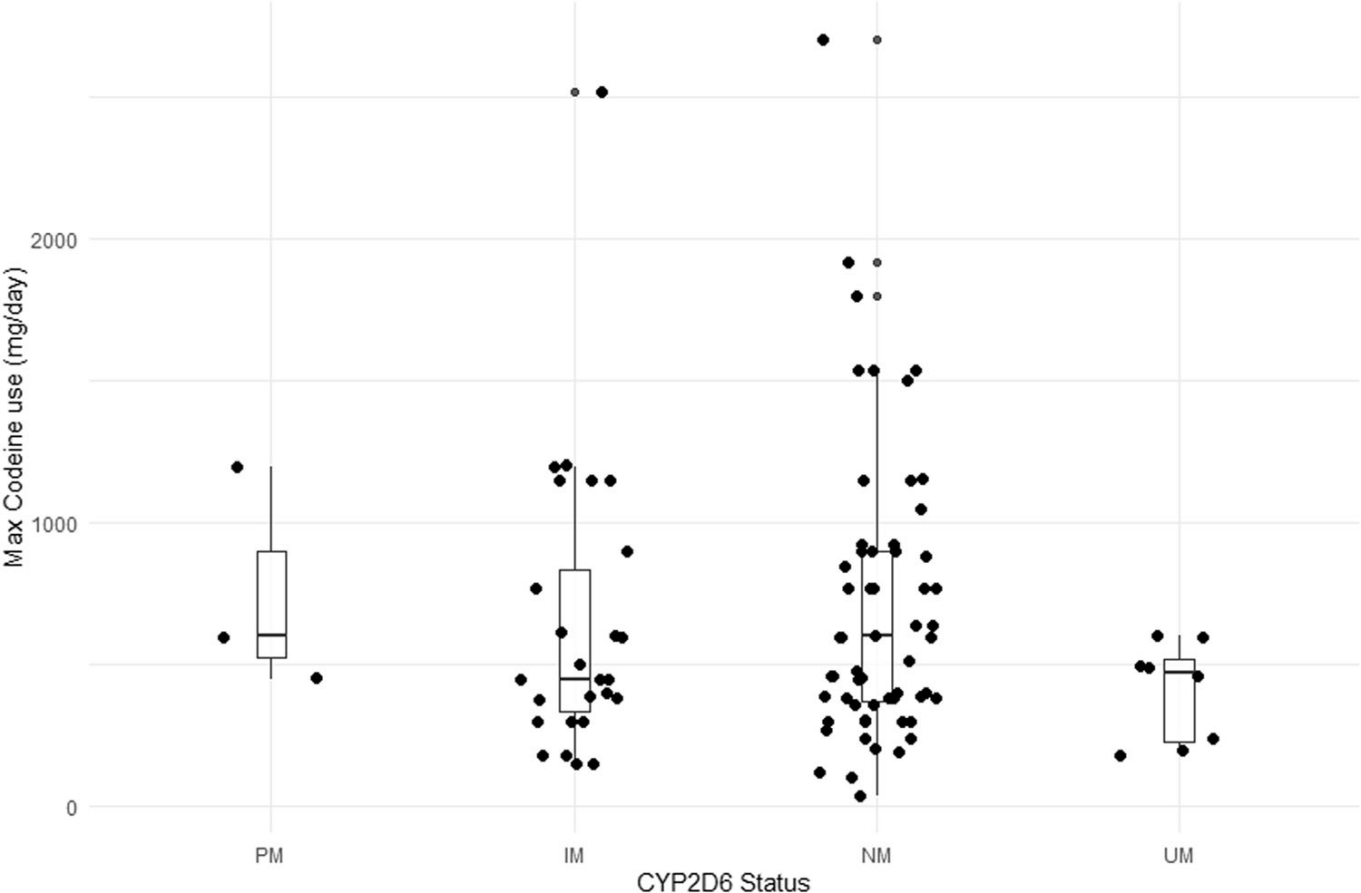
Codeine doses across CYP2D6 phenotypes. PM poor metaboliser, IM intermediate metaboliser, NM normal metaboliser, UM ultrarapid metabolizer.

**Fig. 3:**
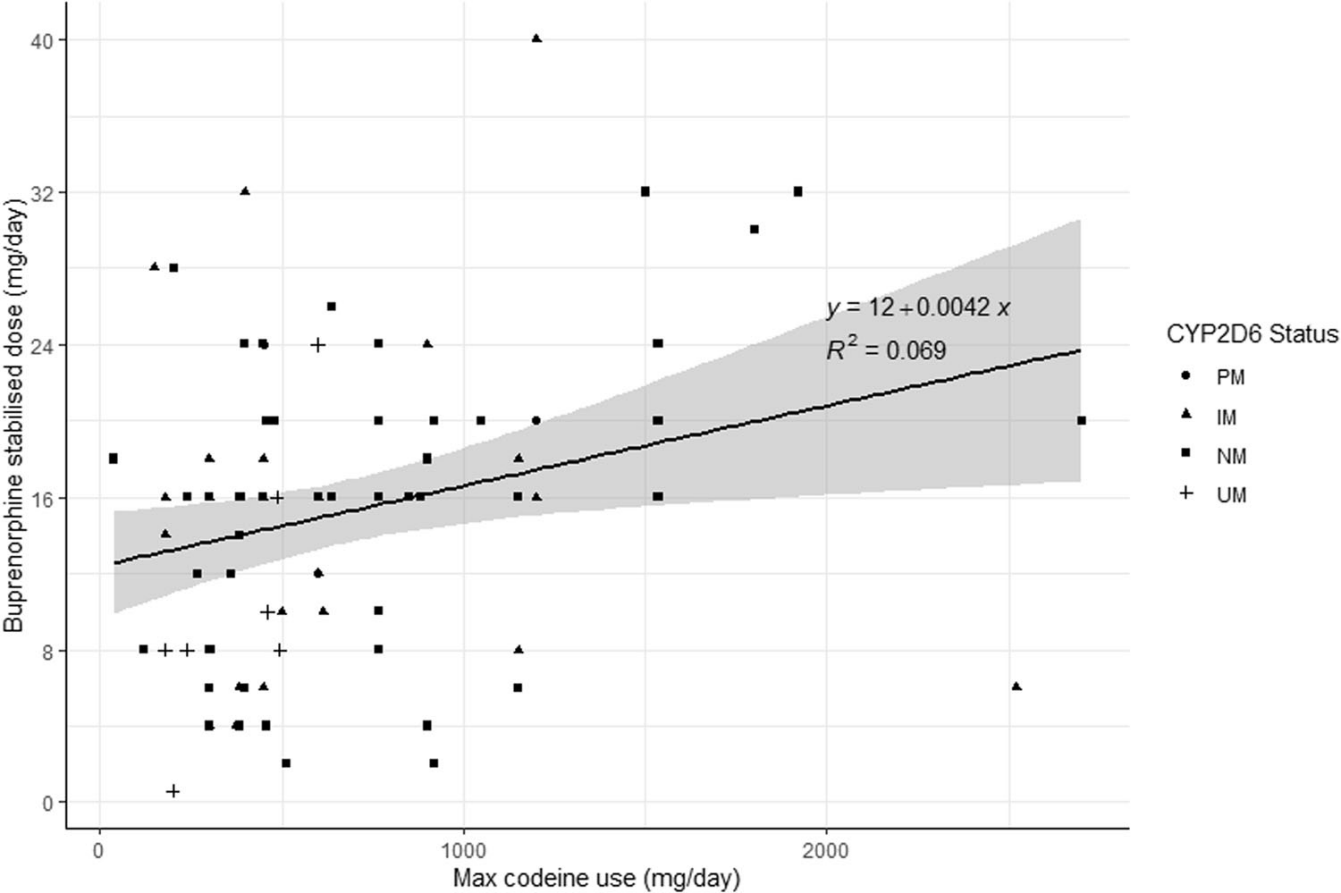
Relationship between stabilisation buprenorphine doses and maximum codeine dose. PM poor metaboliser, IM intermediate metaboliser, NM normal metaboliser, UM ultrarapid metabolizer.

**Fig. 4:**
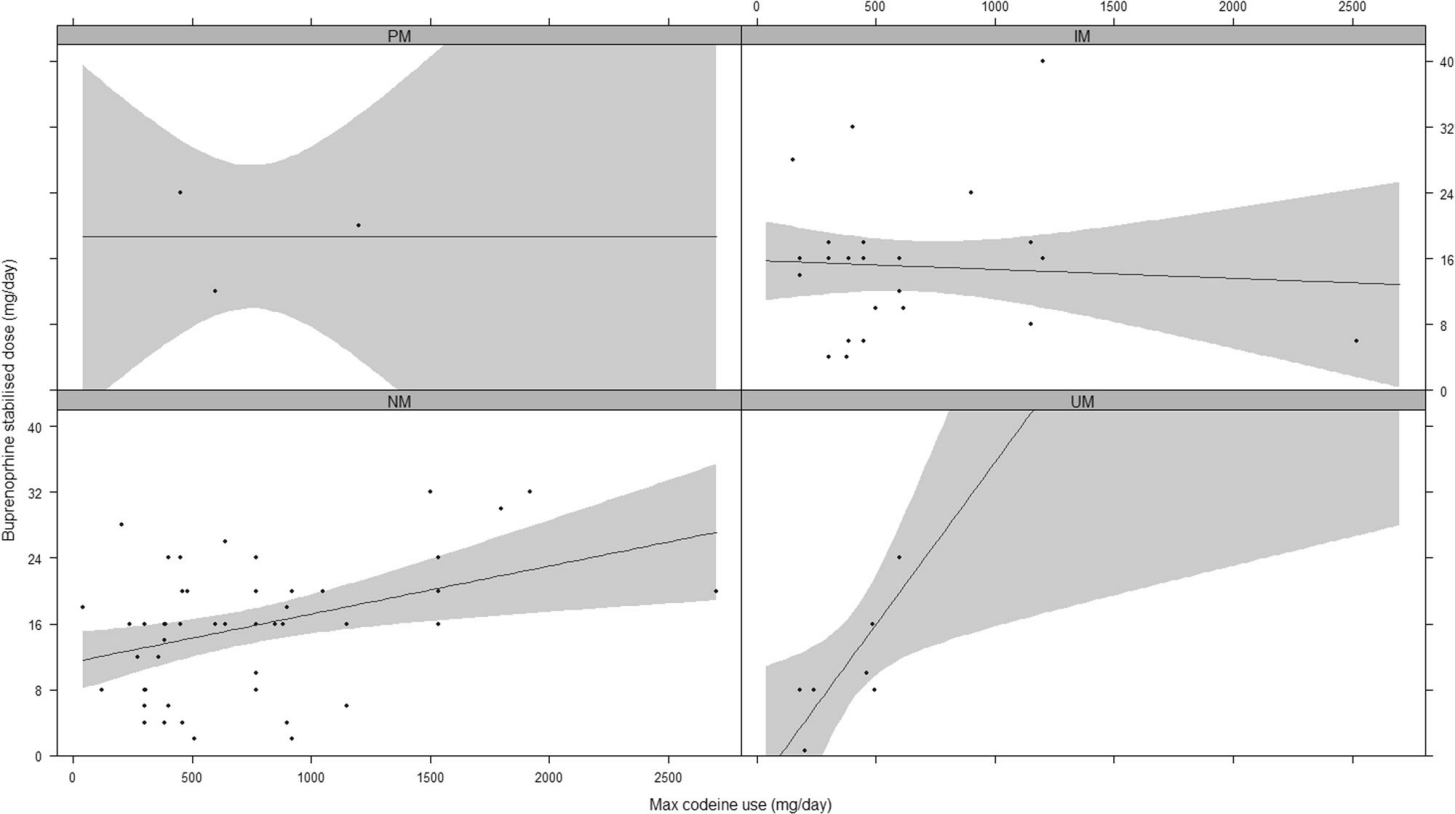
Interaction between codeine and CYP2D6 phenotype on stabilisation dose of buprenorphine. PM poor metaboliser, IM intermediate metaboliser, NM normal metaboliser, UM ultrarapid metabolizer.

There was no statistical difference in codeine doses between CYP2D6 phenotype groups on one-way ANOVA (*F* = 0.88, *p* = 0.46) as shown in Table 1 and Fig. 2. The result was the same using CYP2D6 activity scores instead of phenotype groups (data not shown). As so few participants chose withdrawal or methadone, we excluded these participants from the analyses comparing codeine use to treatment doses, and only included participants who were stabilised on sublingual buprenorphine. Linear regression testing of the main effect of codeine doses pre-treatment on stabilisation doses of buprenorphine demonstrated a statistically significant, but weak, association (*F*_(1,85)_ = 6.34, *p* = 0.01, see Fig. 3). We then added CYP2D6 to the model testing both the phenotype group and the allele activity scores. The model with maximum codeine dose and phenotype group (*F* = 1.78_(4,82)_, *p* = 0.14, adj *R*^2^ = 0.04) was a poor fit and the model with allele activity scores appeared slightly better (*F* = 3.24_(2,84)_, *p* = 0.04, adj *R*^2^ = 0.05) but was not a significantly better fit (*F* = 0.29, *p* = 0.75). We then tested the same models with the addition of an interaction term. Linear regression testing of the interaction between codeine dose and CYP2D6 phenotype group on buprenorphine doses revealed a significant overall model fit (*F*_(7,79)_ = 2.30, *p* = 0.03). The main effect of maximum codeine dose was significant (*t* = 2.06, *p* = 0.04) as was the linear interaction between phenotype and codeine dose (*t* = 1.97, *p* = 0.05), but there was no main effect of phenotype (*t* = −1.81, *p* = 0.07). Individually fitting regression models for each phenotype revealed an apparent progressive increase in the association between codeine and buprenorphine doses with increasing CYP2D6 activity such that the association was non-significant in the PM & IM groups, moderately significant in the NM group (*F*_(1,50)_ = 9.60, *p* < 0.001, Adj. *R*^2^ = 0.14) and most strongly associated in the UM group (*F*_(1,6)_ = 13.06, *p* = 0.01, Adj *R*^2^ = 0.63) as shown in Fig. 4.

Repeating the same test of the interaction between codeine doses and CYP2D6 allele activity score instead of phenotype yielded a model with a reasonable fit (*F*_3,83_ = 3.96, *p* = 0.01, Adj *R*^2^ = 0.09) but no significant difference in model fit compared to the phenotype group analysis (*F* = 1.05, *p* = 0.39). However, the significant predictors of buprenorphine dose were different with the interaction term (*t* = 2.22, *p* = 0.029) and the CYP2D6 activity score (*t* = −2.18, *p* = 0.032) rather than the maximum codeine dose (*t* = −1.32, *p* = 0.189) showing the strongest association.

Smoking status had no association with maximum codeine doses. Adding smoking status to the interaction model for the relationship between codeine doses, buprenorphine doses and CYP2D6

status reduced the model fit and smoking status was not a significant term in the model (data not shown). There were no sex differences in the distribution of CYP2D6 status (data not shown).

## Discussion

The field of pharmacogenomics is rapidly evolving, with results beginning to drive treatment decisions [19]. This study examined the effect of CYP2D6 enzyme phenotype on codeine use disorder and its treatment with buprenorphine. The data supported three of the four a priori hypotheses: there was a shift in proportions of people with CUD towards higher CYP2D6 activity phenotypes; level of daily codeine use was associated with buprenorphine doses required to stabilise; the strength of association between codeine and buprenorphine doses increased with higher CYP2D6 activity phenotype; however, CYP2D6 activity was not associated with codeine usage levels pre-treatment.

This study adds further evidence to existing data that without information on CYP2D6 status, the relationship between daily codeine consumption and buprenorphine doses is statistically significant, but with limited explanatory power or clinical utility [7]. The strong association between codeine and buprenorphine doses in people with ultrarapid CYP2D6 activity suggests that with sufficient conversion of codeine to morphine this becomes the dominant factor in determining ORT doses. However, the wide range of daily codeine usage in the NM group suggests that other factors may be relevant. For example, concomitant use of other common medications which inhibit CYP2D6 [20] could result in phenoconversion to lower levels of CYP2D6 activity [12] tending to diminish the effects of NM and UM status. This was a limitation of the current study as we were unable to record what medications were being prescribed by other services and taken at the time of maximum codeine usage. Approximately 50% of the participants were on other medications (usually psychotropics, mostly antidepressants) at sometime during treatment with ORT. The finding that it was difficult to extract this information from the case notes suggests that clinicians are not investigating the possibility of drug-drug interactions routinely when people present for treatment. These interactions are less relevant to buprenorphine than with methadone or codeine [21], so the co-medications while in treatment are unlikely to have influenced the results of this study, but the lack of information pre-treatment could have modified the effective codeine doses, which may affect the results.

A further limitation of this study was the lack of ethnicity data. This was not collected from the CUD sample and not available for the general population sample. Both samples were taken from Australian clinical services and are therefore likely to have been drawn from the same ethnic distribution. Our general population sample may be subject to some selection bias as they had to be referred for genotyping by a clinician. We therefore cannot exclude some form of ethnic, or other selection bias in the CUD or general population samples that may account for some of the observed differences in allele frequencies. However, this would not account for the gene-drug interaction with codeine and buprenorphine doses, which suggests codeine is likely to be the driver of the observed differences.

Lastly, as we only examined the possible relationship between codeine and buprenorphine in doses associated with CUD and the treatment of CUD with ORT, the relationship between doses of these two medications demonstrated in the results may not be extrapolated to the lower dose ranges normally used for analgesia.

## Conclusions

This study suggests that low CYP2D6 activity may be partially protective against developing a CUD due to the lack of conversion to morphine. If CYP2D6 genotyping becomes part of routine care in the future, it may be useful in predicting risk of toxicity and/or dependence prior to a clinical decision on the prescription of codeine or other opioids (such as tramadol) with more active metabolites. It also highlights that drug-drug interactions are important to consider for illicit and over-the-counter substance use as well as for prescribed medications.

### Supplementary information

Table S1

## Data Availability

The data that support the findings of this study are available on request from the corresponding author. The data are not publicly available due to privacy or ethical restrictions.
